# Supplementary material for: Functional Characterization of Tomato ShROP7 in Regulating Resistance against Oidium neolycopersici
Source: Int J Mol Sci. 2022 Aug 2;23(15):8557. doi: 10.3390/ijms23158557 (PMC9369182; doi:10.3390/ijms23158557)
Supplement: Supplementary file 1 [file ijms-23-08557-s001.zip › ijms-1749324-supplementary.pdf]

**Supplementary Table S1.** DNA primers used in this study.

| Target gene name | Primer name | Sequence (5' to 3') |
|---|---|---|
| Gene cloning | ShROP7-C-F | ATGACTACTGCTAGATTCATCAAATGTG |
| | ShROP7-C-R | TCATAGAATTGCACATAACGTCCTC |
| qPCR analysis | ShROP7-Q-F | CCAACACTCACCACTACACCTACTT |
| | ShROP7-Q-R | CATTGCTTACACCACCCTTTTTACT |
| | ShGAPDA-Q-F | CTGGTGCTGACTTCGTTGTTG |
| | ShGAPDA -Q-R | GCTCTGGCTTGTATTCATTCTCG |
| | ShPR1-F | TCACAATGCAGCTCGTAGACAAGTT |
| | ShPR1-R | TTCACCGTAAGGTCCACCAGAGT |
| | ShPR5-F | CCTACACCGTTTGGGCAGCAT |
| | ShPR5-R | GCATGAACCTCTACCAGCACCATTA |
| | ShPDS1.2-F | TGAATGGCACAGCAGTTGGTTCT |
| | ShPDS1.2-R | TGTTCCATAATAGCAGCAGCCTCAA |
| VIGS | ShROP7-V-F | agaaggcctccatggggatccATGTGGTGGTGGATGGTAGCA |
| | ShROP7-V-R | cgtgagctcggtaccggatccAGGAGTAGCCCCTGGATGATCA |
| | ShPDS-V-F | gtaaggttaccgaattctctagaCAAGCAAGCCAGGAGAAT |
| | ShPDS-V-R | tcgggacatgcccgggcctcgagGCAAGTGTGATGTCCATAGTAT |
| | ShROP7-S-F | GCTCCAAGACTCAGCAGAATG |
| | ShROP7-S-R | TTTCTTCATCTTAGGGGGTCG |
| Overexpression in tobacco | ShROP7-O-F | agcatcgattcccgggtcgacATGACTACTGCTAGATTCATCAAATGTG |
| | ShROP7-O-R | aaccgttcatcggcggtcgacTCATAGAATTGCACATAACGTCCTC |
| Y2H | ShROP7-B-F | aggccgaattcccggggatccATGACTACTGCTAGATTCATCAAATGTG |
| | ShROP7-B-R | ccgctgcaggtcgacggatccTCATAGAATTGCACATAACGTCCTC |
| BIFC | ShROP7-cY-F | tggcgcgccactagtggatccATGACTACTGCTAGATTCATCAAATGTG |
| | ShROP7-cY-R | gacagtactatcgatggatccTAGAATTGCACATAACGTCCTC |
| | ShSOBIR1-nY-F | cccaggcctactagtggatccATGACCTTCACAGCCTCATATATACAC |
| | ShSOBIR1-nY-F | accctcgaggtcgacggatccTTAATGCTTGATCTGCATCAACATG |

Note: The primer sequence is composed of two parts. The lowercase letters represent the sequences of corresponding vector homologous arm and restriction enzyme site, and the capital letters indicate the gene-specific nucleotide sequences.
